# Draft genome sequence of *Pseudomonas aeruginosa* strain maqsudiensis isolated from cattle swab in Dhaka, Bangladesh

**DOI:** 10.1128/mra.01190-24

**Published:** 2025-05-21

**Authors:** Md. Mashiur Rahaman, Abdus Sadique, Jahidul Alam, Fahad Khan, Zinat Ayesha, Shoriful Islam, Monsur Murshid, Raiyan Rafsan, Shariful Islam, Humaira Binte Iqbal, Taposh Kumar Das, Oly Ahmed, Kamruzzaman Rumman, Ayesha Sharmin, Md. Mainul Hossain, Munirul Alam, Maqsud Hossain

**Affiliations:** 1 NSU Genome Research Institute (NGRI), North South University 54495 , Dhaka, Bangladesh; 2 Department of Biochemistry & Microbiology, School of Health and Life Sciences, North South University, Dhaka, Bangladesh; 3 Department of Electrical and Computer Engineering, School of Engineering & Physical Sciences, North South University, Dhaka, Bangladesh; 4 Popular Diagnostic Centre, Dhaka, Bangladesh; 5 Invent Technologies Limited, Dhaka, Bangladesh; 6 National Institute of Cancer Research and Hospital (NICRH) 332897 , Dhaka, Bangladesh; 7 Department of Chemistry, Bangladesh University of Engineering & Technology, Dhaka, Bangladesh; 8 icddr,b, International Centre for Diarrheal Disease Research, Dhaka, Bangladesh; The University of Arizona, Tucson, Arizona, USA

**Keywords:** draft genome, *Pseudomonas aeruginosa*

## Abstract

Here, we present the draft genome sequence of *Pseudomonas aeruginosa* strain maqsudiensis, isolated from cattle stool swab. The genome was sequenced using the Illumina MiSeq platform, yielding a 6.33 Mb assembly with 5,784 predicted coding sequences. The genome harbors multiple antibiotic-resistance genes and virulence factors, providing insights into *P. aeruginosa* colonization in livestock and potential implications for antimicrobial resistance dissemination through the food chain.

## ANNOUNCEMENT

*Pseudomonas aeruginosa* is a highly adaptable opportunistic pathogen found in diverse environments, including animal hosts (1). Understanding its presence in livestock populations is crucial for monitoring potential zoonotic transmission and tracking antimicrobial resistance patterns in food animals.

Strain maqsudiensis was isolated from cattle rectal stool swabs collected at Gabtoli Cattle Market (23.7821°N, 90.3374°E) on 13 September 2021, the largest livestock market in Dhaka, Bangladesh. The samples were collected from cattle rectal stool using sterile swabs and were initially inoculated in LB broth and grown overnight at 37°C for enrichment and then cultured on MacConkey agar plate at 37°C for 18–20 h. Species identity was confirmed through colony morphology and PCR with species-confirming primer (2). Genomic DNA was extracted from a single colony grown overnight at 37°C in LB broth using the QIAGEN Mini Kit (QIAGEN, Germany) following the manufacturer’s protocol, and its purity was confirmed with a NanoDrop 2000 Spectrophotometer (Thermo Fisher Scientific, USA). Library preparation was performed using the NEBNext Ultra II DNA Library Prep Kit (New England Biolabs, USA) following the manufacturer’s protocol. The whole-genome sequencing was conducted on an Illumina MiSeq platform, generating 2 × 150 bp paired-end reads. The sequencing run produced 637,121 paired-end reads. Quality control was performed using FastQC v0.11.9 (3), and reads were trimmed using Trimmomatic v0.39 (AVGQUAL 30) (4) to remove low-quality sequences and adapters. The resulting 464,433 high-quality reads were assembled using Unicycler v0.4.8 (5) with default parameters. Quality assessment using Quast 5.2.0 (6) revealed that the final assembly consists of 103 contigs with a total length of 6,337,618 bp and a GC content of 66.44%. The *N*_50_ value is 106,230 bp, with the largest contig being 436,147 bp. Annotation of the assembled genome was done locally by Prokka v1.14.6 (7) and the Prokaryotic Genome Assembly Pipeline (PGAP) v6.8 (8) by NCBI. All tools were run with default parameters unless mentioned otherwise.

Prokka identified 5,784 coding sequences (CDS), 3 rRNA genes, 65 tRNA genes, and 1 tmRNA. Meanwhile, PGAP predicted a total of 5,944 genes, of which 5,847 are protein-coding genes along with 3 rRNA and 56 tRNA. Analysis using the ResFinder server v4.6.0 (9, 10) revealed multiple antibiotic-resistance genes, including *crpP*, *blaPAO*, *blaOXA*, *catB7*, *aph(3′)-IIb*, *and fosA*. Additionally, PathogenFinder v1.1 (11) analysis indicated a probability of 0.759 for human pathogenicity, suggesting potential zoonotic concerns.

To the best of our knowledge, this genome sequence represents the first *P. aeruginosa* isolate from cattle in Bangladesh to be fully sequenced, providing valuable insights into the genomic features of livestock-associated strains. The presence of multiple antibiotic-resistance genes in this isolate highlights the potential role of livestock as reservoirs for antimicrobial-resistant *P. aeruginosa* strains.

## Data Availability

The sequence reads have been deposited in the NCBI Sequence Read Archive under accession number SRR31155591. The associated BioProject and BioSample accession numbers are PRJNA1179666 and SAMN44500193, respectively. The whole-genome shotgun project has been deposited at DDBJ/ENA/GenBank under accession number JBITND000000000.
